# Identification of Specific Effect of Chloride on the Spectral Properties and Structural Stability of Multiple Extracellular Glutamic Acid Mutants of Bacteriorhodopsin

**DOI:** 10.1371/journal.pone.0162952

**Published:** 2016-09-22

**Authors:** Tzvetana Lazarova, Krzysztof Mlynarczyk, Enric Querol, Boris Tenchov, Slawomir Filipek, Esteve Padrós

**Affiliations:** 1 Unitat de Biofísica, Departament de Bioquímica i de Biologia Molecular, Facultat de Medicina, and Centre d'Estudis en Biofísica, Universitat Autònoma de Barcelona, Barcelona, Spain; 2 Faculty of Chemistry, Biological and Chemical Research Centre, University of Warsaw, Warsaw, Poland; 3 Institut de Biomedicina i Biotecnologia, Universitat Autònoma de Barcelona, Barcelona, Spain; 4 Department of Medical Physics and Biophysics, Faculty of Medicine, Medical University – Sofia, Sofia, Bulgaria; Universitat Politecnica de Catalunya, SPAIN

## Abstract

In the present work we combine spectroscopic, DSC and computational approaches to examine the multiple extracellular Glu mutants E204Q/E194Q, E204Q/E194Q/E9Q and E204Q/E194Q/E9Q/E74Q of bacteriorhodopsin by varying solvent ionic strength and composition. Absorption spectroscopy data reveal that the absorption maxima of multiple EC Glu mutants can be tuned by the chloride concentration in the solution. Visible Circular dichroism spectra imply that the specific binding of Cl^-^ can modulate weakened exciton chromophore coupling and reestablish wild type-like bilobe spectral features of the mutants. The DSC data display reappearance of the reversible thermal transition, higher T_m_ of denaturation and an increase in the enthalpy of unfolding of the mutants in 1 M KCl solutions. Molecular dynamics simulations indicate high affinity binding of Cl^-^ to Arg82 and to Gln204 and Gln194 residues in the mutants. Analysis of the experimental data suggests that simultaneous elimination of the negatively charged side chain of Glu194 and Glu204 is the major cause for mutants’ alterations. Specific Cl^-^ binding efficiently coordinates distorted hydrogen bonding interactions of the EC region and reconstitutes the conformation and structure stability of mutated bR in WT-like fashion.

## Introduction

One of the challenging questions in molecular biology is how membrane proteins fold and assemble *in vivo* in biologically active structures. In the last decade has been substantial progress towards understanding the molecular nature of the forces governing the structural stability of the membrane proteins and the contributions of these forces for protein functioning [1]. Important elements of the molecular machinery required for conformational stability of the membrane proteins have been elucidated, yet further advancement is needed to resolve completely this enigmatic process [2–5]. Exploring this question is important not only for better understanding how the unique structural and functional integrity of the protein is achieved and maintained, but also to improve our knowledge on how proteins are driven into misfolded conformations in disease states.

The light driven pump bacteriohodopsin (bR) is a membrane protein, which transports a proton out of the cell upon illumination, thus establishing a proton gradient for energy generation. In a close structural relationship to G-protein coupling receptors, this protein consists of seven α helices, embedded into the plasma membrane of *Halobacterium salinarum*. One of the defining features of bR is the retinal chromophore, which is covalently bound to the polypeptide chain via a Schiff base (SB) to a Lys residue. The photoisomerization of the retinal triggers a series of protein conformational changes, which in turn make the proton pump working. Certainly, the biological activity of bR depends on the retinal, which serves as a *ligand* for unphotolysed bR similarly to other signal transduction proteins. In *vivo* bR molecules are assembled into trimers, arranged as a two-dimensional (2D) hexagonal lattice, known as purple membrane (PM) [6].

A number of previous studies have established that specific protein-chromophore, protein-protein and protein-lipid interactions are involved in the conformational dynamics, structural stability and arrangement of bR [7–9]. Influence of specific lipid—protein contacts on the paracrystalline lattice formation has been first illustrated by the recovery of 2D arrays in detergent-solubilized bR monomers, mixed with bR membrane lipids [10]. The high resolution maps of bR have provided further insight into the involvement of PM lipids into lattice formation through visualization of specific contacts between charged lipids, placed into the bR trimers interior, and the surrounding protein [11–13].

It is well known that the purple membrane, treated with hydroxylamine, and retinal-deficient strains lack the characteristic 2D array, but the addition of retinal regenerates bR structurally [14–16]. Furthermore, AFM studies made clear that the transformation of bR into bacterio opsin strongly affects trimer interactions [17]. All these studies pointed out that retinal-protein interactions have critical role not only for protein folding, but also for the lattice formation [18,19]. Basically, mutational studies have provided information on the importance of some inter-and intra-molecular interactions on the stability and 2D packing of bR [20,21]. Currently, the critical role of hydrogen-bonded water network for providing proton transfer pathway of bR is well established. The active center of unphotolysed bR is defined by the highly polarized water molecule, hydrogen bonded to the protonated SB and to two anionic aspartates [22]. Changes in hydrogen bonding interactions of key residues have been shown to affect the active site, as well as the structural stability of whole bR [23,24].

Taken together, these studies testify that the conformational stability and 2D arrays of bR are a result of a complex makeup, in which chromophore-protein, lipid- protein, and protein-protein interactions are all essential factors. Despite of the immense amount of existing data, the question whether distortion of the continuous hydrogen bond network in the extracellular (EC) side can provoke disordering of paracrystalline array of bR monomers in PM is not sufficiently explored and remains unclear.

Previously, the glutamate residues Glu9, Glu74, Glu194 and Glu204, located on the EC side of the bR were subject to functional studies with the objective to resolve the proton release mechanism [25]. Our earlier DSC studies reported that site–directed mutagenesis of Glu9, Glu204 and Glu194 for Gln affects the temperature of bR unfolding, suggesting the involvement of these Glu side chains in maintenance of the protein stability [26]. Furthermore, we found that simultaneous multiple mutations of EC Glu residues for Gln, such as double E204Q/E194Q (2Glu), triple E204Q/E194Q/E9Q (3Glu) and quadruple E204Q/E194Q/E9Q/E74Q (4Glu) reduce bR intrinsic stability and, furthermore, disordering of the 2D array, as seen by the lack of DSC pre transition in these mutants in water [24,26,27].

Here we extended our studies of these multiple EC Glu mutants aiming to advance our understanding of the molecular mechanisms that govern the 2D assembly and stability of bR. By using different spectroscopic methods and DSC, we provide experimental evidence that the specific binding of chloride to multiple EC Glu mutants results in the restoration of WT-like conformation and paracrystalline array of mutated proteins. Furthermore, molecular dynamics (MD) simulations provide a molecular glance on how chloride restores WT-like behavior, revealing its specific binding to Arg82, and to Gln204 and Gln194 residues in the mutants.

## Materials and Methods

### Construction of Multiple Extracellular Glu Mutants

Construction of 2Glu (E194Q/E204Q), 3Glu (E9Q/E194Q/E204Q) and 4Glu (E9Q/E194Q/E204Q/E74Q) mutants was accomplished by cloning of the single mutants together, taking advantage of unique restriction sites. After screening of the mutant by DNA sequencing, it consequently was transformed and expressed in the *H*. *salinarum* L33 strain. The purple membranes were grown and purified, following the standard procedure as previously described [28].

### UV-VIS Absorption Spectroscopy

UV-VIS absorption spectra between 250 and 750 nm were recorded on a Varian Cary3Bio spectrophotometer, supplied with integrating sphere to avoid light scattering artifacts. For light adaptation of the samples the illumination was carried for 10 min, using a light projector with a cutoff filter (>520 nm). Because of the abnormal thermal isomerization of the mutants, dark-adapted samples were kept in the dark at least for 4 weeks at room temperature [29].

For Cl^-^ titration experiments, small aliquots of resuspended purple membranes in water, containing mutated bacteriorhodopsin (8 μM) were mixed with solutions containing KCl, at various concentrations (0–2 M). The samples were first dark-adapted, then transferred to 3 ml quartz cuvette and the spectra from 800 nm to 250 nm were recorded at room temperature, pH 6.0. Alkaline titration experiments were carried out with dark-adapted samples in 150 mM and 1 M KCl, at room temperature. The pH was adjusted by the addition of small aliquots of NaOH and then the absorption spectra were measured. The difference spectra were calculated by subtracting the absorption spectrum taken at different pH from the absorption spectra at pH 6.0. We noted that mutated proteins were more prone to aggregation, especially at elevated pH, where a gradual loss of absorbance was observed. The experimental data were fitted by the Henderson-Hasselbach equation using the OriginLab software.

### Extraction of the Retinal and HPLC Analysis

The procedure of the retinal extraction and HPLC analysis was as described previously [29]. Dark and light adaptation of the samples was performed as in UV–Vis experiments.

### Circular Dichroism

CD studies were performed on a JASCO J700 CD spectrometer, supplied with Peltier temperature controller. All CD spectra were recorded using 0.1 or 1 cm quartz cuvettes and all spectra were obtained after averaging at least of eight runs. Thermal scans were run at a scanning speed of 1.5 K/min in the temperature range between 293.15 and 371.15 K. The samples were allowed to equilibrate for 8 min at each temperature. The ellipticity changes at 530 and 460 nm as a function of temperature were used for the analysis of thermal transitions. The thermal traces were fitted with a single sigmoid Boltzmann equation to yield mid-transition temperatures, T_m_. The data for WT fit better to a double sigmoid function, using two thermal ranges, based on the analysis of the variance and the residuals.

### Differential Scanning Calorimetry (DSC)

DSC experiments were performed using a Nano DSC instrument from TA Instruments, USA. Prior to DSC scans, solutions of wild type and mutant bRs were prepared by exhaustive dialysis against water or 1 M KCl, pH 7.0. Before the measurements, the sample and reference solutions were properly degassed and carefully loaded into the cell to avoid bubble formation. Subsequently, the samples were introduced into DSC cell holder with a final concentration in the range 1.3–2 mg/ml of bR (the protein concentrations were determined spectrophotometrically). The reference cell was loaded with the aqueous medium obtained from the dialysis of the purple membrane suspension. Data were collected in the temperature range from 298.15 to 383.15 K at a heating rate of 1K/min. After the first heating scan was completed, the samples were cooled to 25°C, and reheated to check transitions reversibility. For each sample, consecutive thermograms were recorded. The DSC data were processed and analyzed using NanoAnalyse software, following the Takahashi and Sturtevant methodology, as previously explained [24].

### Molecular Dynamics Simulations

Two sets of simulations were performed, using different chloride concentration: 2 M for investigation of chloride entry path and 0.15 M for checking the stability of ion’s binding position as predicted with Yasara [30]. All simulations were performed and analyzed using Gromacs suite [31]. R statistical computing software [32] was used for data processing. VMD [33] was the primary tool for trajectory visualization, while the figures were obtained using PyMol [34]. Details (system composition and the simulation setup) are largely identical to our previous study [24] and described in S1 Appendix.

## Results

### Chloride-Specific Spectral Properties of Unphotolysed Multiple EC Glu Mutants

#### Color Regulating Effect of the Chloride anion

**Fig 1:**
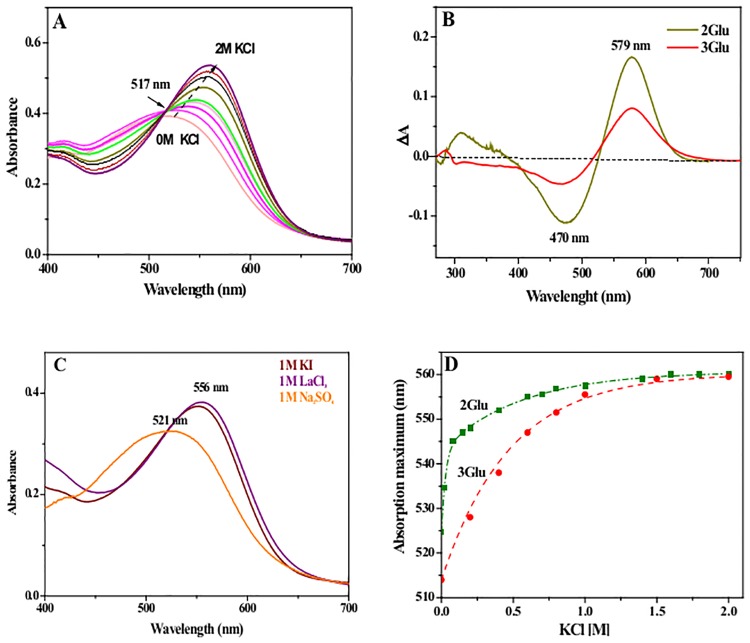
Effects of the ionic strength and the composition of the media on the UV/ VIS absorption spectra of multiple EC Glu mutants. (A) Representative absorption spectra of 3Glu mutant in KCl concentrations ranging from 0 to 2 M, pH 6.0, 25°C. The arrow indicates the direction of absorption maximum changes with increasing salt concentration. The isosbestic point at 517 nm indicates that a transition between only two spectral species, purple and red, is taking place. (B) Difference spectra of 2Glu and 3Glu, calculated by subtraction of an absorption spectrum, recorded in 1 M KCl minus a spectrum in water, pH 6.0, 25°C. The difference spectrum of 4 Glu (not shown) shows similar spectral features [35]. (C) Absorption spectra of 3Glu in 1 M KI, 1 M LaCl_3_ and 1 M Na_2_SO_4_ at pH 6.0, 25°C. (D) Plots of the absorption maxima (λ_max_) of dark-adapted samples of 2Glu and 3Glu vs KCl concentration. Dotted lines represent the best nonlinear curve fits using the Hill1 equation (OriginLab software) yielding the apparent dissociation constants.

Unlike the single EC Glu mutants (E194Q, E204Q, E9Q and E74Q) the spectral properties of the double, 2Glu, the triple, 3Glu and the quadruple, 4Glu mutants are highly dependent on the ionic strength of the media. In water and in low ionic strength, at neutral pH, the visible absorption maxima (λ_max_) of 2Glu (~531 nm), 3Glu (~ 517 nm), and 4Glu (~510 nm) are strongly blue-shifted, compared to WT bR (568 nm) maximum. As we reported previously the spectral blue shift of the absorption maxima of multiple EC mutants at neutral pH is caused by the presence of considerable amount of species, absorbing around 460 nm, so called “red” form species [24, 35,36]. The titration with KCl causes a rising red shift of mutants’ λ_max_, which finally get to values close to the absorption maximum of WT (Fig 1A) [35,36]. These spectral changes are reversible and with the elimination of KCl from the media, the λ_max_ go back over blue spectral shifts, consequently the pink color of the mutated proteins gets recovered. KCl affects not only the visible, but also the UV range of the spectra. Fig 1B shows difference spectra, calculated by subtraction of spectra recorded in presence and in absence of KCl. In the visible range the negative band at ~470 nm illustrates the disappearance of the “red” species at the expense of the purple form 579 nm (+), while small UV bands at ~300 nm and at ~276 nm suggest that the ion binding most likely results in electrostatic perturbation of some Tyr and Trp residues [37]. To clarify whether these spectral alterations are due to specific binding of the Cl^-^ anion or are result of shielding of protein surface charges, we recorded the spectra in the presence of different salts (Fig 1C). We found that Cl^-^ and I^-^ halide anions acts similarly, while SO_4_^2-^ anion fails to show any effect on mutants’ spectra. These findings suggest that the ionic size is critical and most likely halide anions binds to specific site(s) within the mutated bRs.

Next, we performed spectroscopic titrations, recording the absorption spectra at different concentration of KCl to examine the binding affinity of Cl^-^ for the mutants. Plots of the λ_max_ vs KCl concentration are fitted with non-linear curves yielding apparent dissociation constants (K_d_) of 0.16 ± 0.06, 0.38 ± 0.1 and 0.22 ± 0.12 M KCl for 2Glu, 3Glu and 4Glu [36], respectively (Fig 1D).

#### The Schiff Base pKa

**Fig 2:**
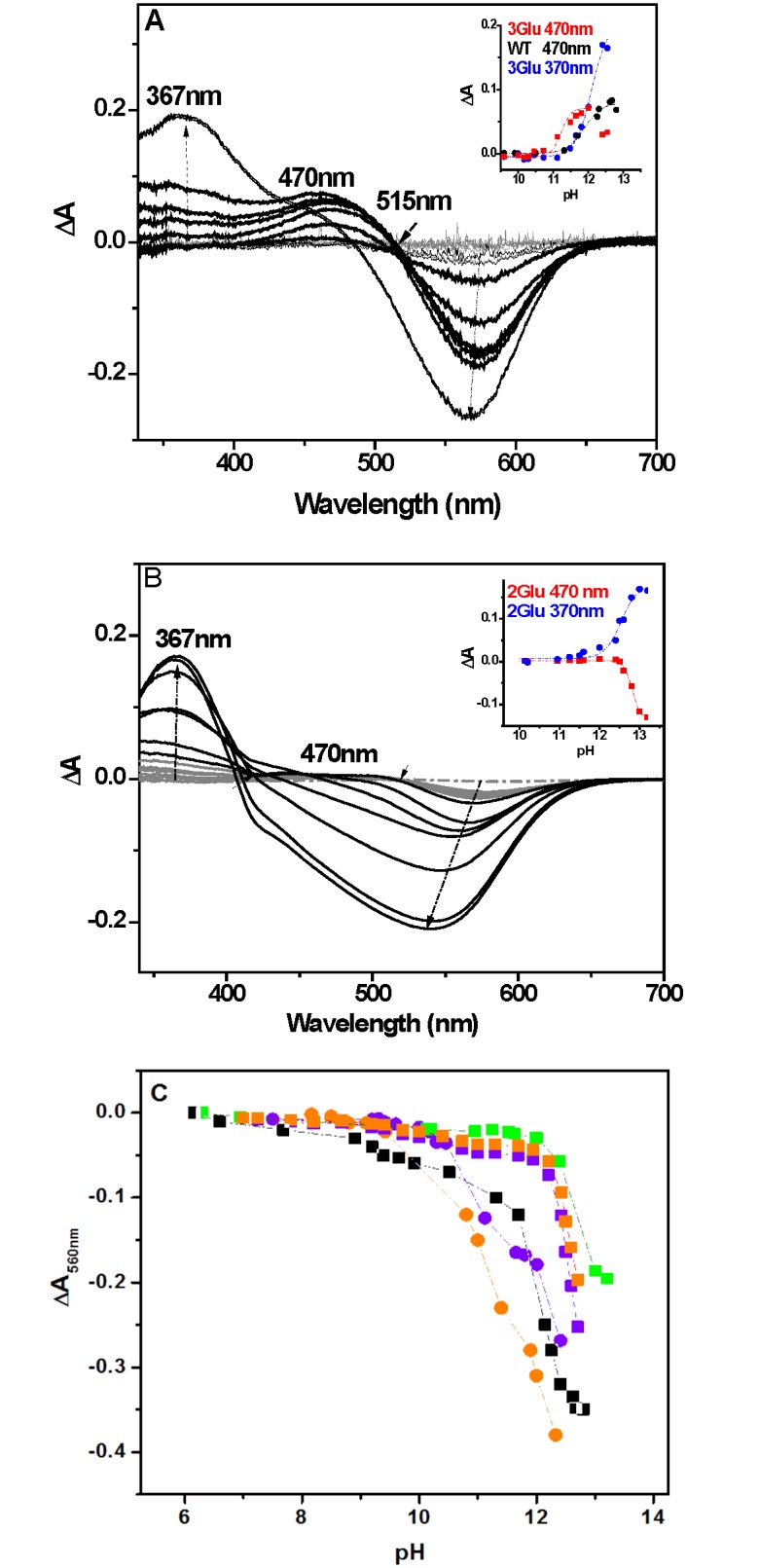
Alkaline titration of the Schiff base of multiple EC Glu mutants and WT. (A) Representative difference spectra (pH_i_–pH_6_) of 3Glu in 1 M KCl (for pH values i = 9 to 12.5), at room temperature. The cross over point at 515 nm reflects the transition from the basic (purple in 1 M KCl) to red spectral form. (B) Representative difference spectra (pH_i_–pH_6_) of 2Glu in 150 M KCl (for pH values i = 6 to 13.2), at room temperature. Insets in A and B represent plots of the absorption changes at 470 nm and 370 nm, as a function of the pH. The apparent pKa of SB, at 12.8 and 12.3 for 2Glu (150 mM KCl) and 3Glu (1 M KCl), respectively, were obtained from the best fit of ΔA at 370 nm vs pH using the Henderson-Hasselbach equation. (C) Plots of the absorption changes at 570 nm as a function of pH,for WT (■) and 2Glu (■) in 150 mM KCl, and 3Glu (■, ●) and 4Glu (■, ●) in 150 mM and 1 M KCl, respectively, at room temperature. Continuous lines are empirically drawn for a visual help and do not represent a fit of the data.

The retinal interactions with the residues forming the RBP (retinal binding pocket) are crucial for the absorption maximum of bR [24]. Therefore, it is reasonable to assume that if Cl^-^ resides near the RBP complex, this should affect the Schiff base pKa. To test this scenario we performed alkaline titrations of EC Glu mutants. Fig 2A and 2B show the difference spectra of 3Glu and 2Glu in the presence of high and low salt concentrations. In 1 M KCl the alkaline spectral transitions of the mutants (Fig 2A) are similar to those identified in the WT [37,38]. The increase of pH causes a decrease in intensity of the main absorption maximum, first giving rise to a band at about 470 nm, due to the partial transformation of the pigment into the “red” form, like in the WT [37]. At higher pH, a band at about 367 nm appears, attributed to the retinal release (Fig 2A) [37]. Finally, above pH 12 the latter band becomes dominant, implying the SB hydrolysis and the denaturalization of the protein [24,38].

At low salt concentrations the difference spectra illustrate different titration behavior (Fig 2B). Basically, the spectra consist of one negative band which blue shifts upon pH increase, and one positive band at about 367 nm. The positive band at 470 nm is practically missing, displaying only negligible intensity rise upon alkalization. Furthermore, the half-width of the negative band, reflecting the disappearance of the ground-state spectral forms (obtained at neutral pH) is notably broader with respect to the difference spectra in 1M KCl. In fact, these spectral differences can be accounted for the considerable amount of “red” species present in the mutants at low salt concentrations and neutral pH, as we described above.

Plots of absorption changes at the wavelength of 480 vs pH allow following the formation of the red species, while plots of changes at 370 nm follow the deprotonation of the SB and the retinal release, thus determining the pKa of the SB and the denaturation of the protein. Previous studies of alkaline purple to red transitions in WT [37] reported the formation of two “red” fractions with pKa ~9 (1%) and pKa ~12 (94%). Similarly to WT, in 1 M KCl, the intensity increases of the 480 nm band above pH 10.5, implies for the formation of the red form species in the mutant, however at lower pH than of WT (Fig 2A, inset). In contrast, in 150 mM KCl (Fig 2B, inset) the plot of the absorption at 480 nm vs pH does not show any rise in intensity with the alkalization.

The plots of the absorption changes at 570 nm give better visualization of the disappearance of the ground state spectral forms in multiple Glu mutants upon alkalization in 150 mM and 1 M KCl (Fig 2C). A comparison of the alkaline titration plots reveals differences in the curve shapes between low and high salt conditions. In 1 M KCl, the accumulation of red form species contribute to the amplitude of 570 nm band with pH increases. However, in 150 mM KCl, because of a minor formation of red species, such an effect on the titration curves hardly can be appreciated. Interestingly at low salt concentration the multiple EC Glu mutants imply for a higher stability of the SB to alkaline denaturation, with pKa values about 0.5 pH units higher with respect to the pKa of the mutants in the presence of high KCl concentration.

#### Light-Dark Adaptation

**Fig 3:**
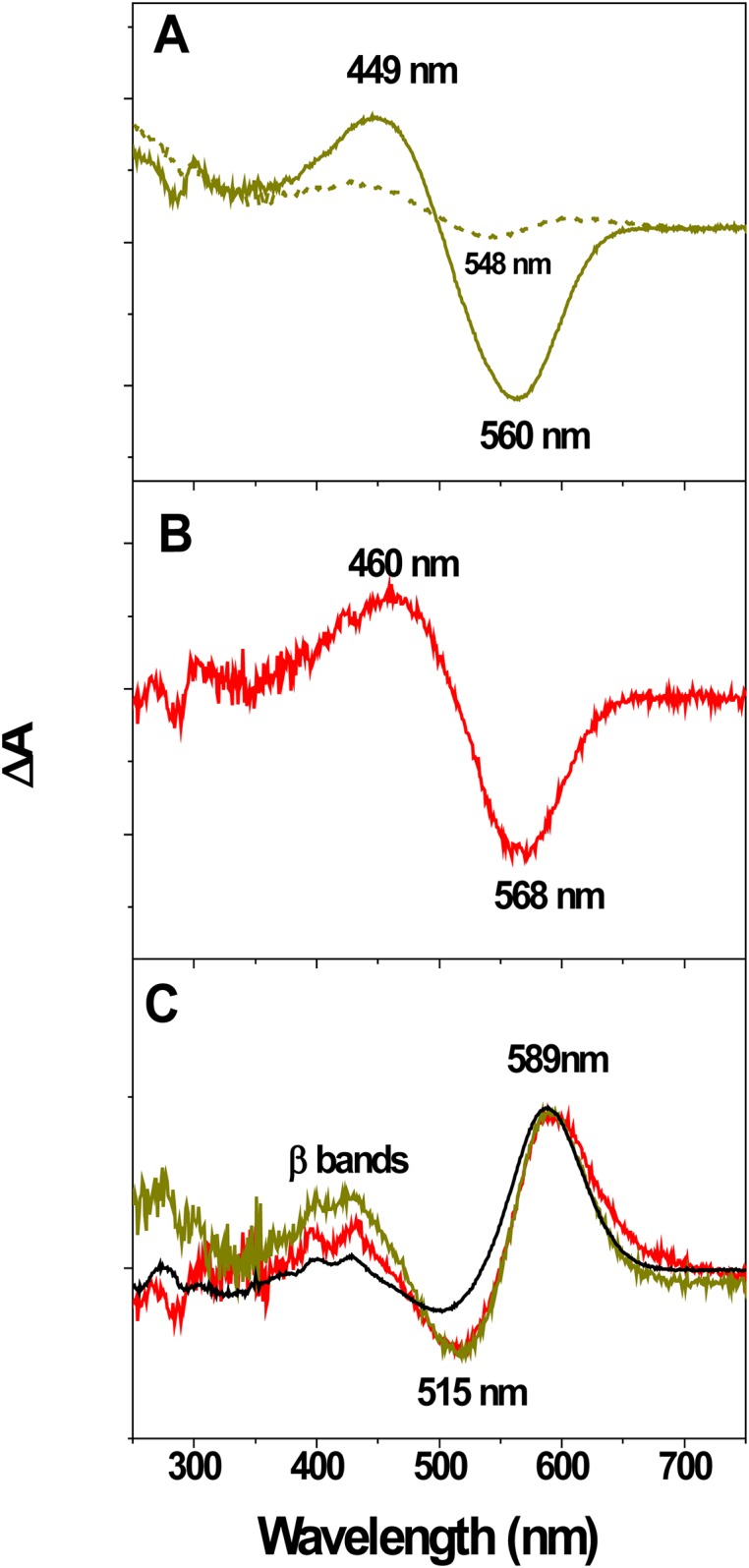
Light minus dark (LA–DA) absorption difference spectra of 2Glu and 3Glu at pH 6.0, room temperature. (A) LA–DA difference spectra of 2Glu in 150 mM KCl (the dotted line corresponds to 0.02 M KCl). (B) LA–DA difference spectra of 3Glu in 150 mM KCl. (C) LA–DA difference spectra of 2Glu (green line) and 3Glu (red line) in 1.5 M KCl. For comparison the difference spectrum of WT (black line) is also plotted. The difference spectra of 4Glu (not shown) [26, 35], recorded in the presence of low and high concentrations of KCl are identical to those of 3Glu.

At room temperature and in the dark, WT contains two retinal isomers: all-*trans*, 15-*anti* and 13-*cis*, 15-*syn* at ratio of 1:2, called dark-adapted bR (bR_DA_). Upon illumination, the retinal configuration turns entirely to the *all-trans* isomer, so called light-adapted bR (bR_LA_) [39]. The rate of light to dark adaptation in the single EC Glu mutants, except for E194Q is similar to that of WT, in contrast to the multiple Glu mutants, which suffer drastically slow thermal isomerization, most likely due to the mutation of Glu194 for Gln [29]. As we reported previously, the presence of chloride in the media modulates not only the position of the absorption maxima, but also thermal *13-cis to all-trans* isomerization of multiple Glu mutants [29,35]. To understand at molecular level the effect of Cl^-^ on the isomerization, we examined the difference spectra between light- and dark-adapted (LA-DA) samples in the range 0 to 2 M KCl. The difference spectra revealed the lack of *all-trans* ß bands, indicative for *cis* to *trans* thermal isomerization in WT, in all salt concentration range up to 1M KCl. Representative LA-DA spectra for 2Glu and 3Glu mutants in presence of low (150 mM) and high (> 1 M) concentrations of KCl are shown on Fig 3. LA-DA difference spectra of samples, containing low concentration of KCl, display a positive band at about 450–460 nm, a negative band at about 548–560 nm and 285 (-)/310 (+) nm pair bands in the near UV range (Fig 3A and 3B). The negative band at 548 nm resembles the absorption maximum of *13cis* bR, while the positive band at 460 nm is characteristic for the “red” spectral form of WT [40,41]. These spectral features suggest that at low salt concentration the illumination triggers purple to “red” spectral-like transition in the mutants, which apparently goes along with conformational changes, leading to perturbation of some aromatic residues. From other side, the absence of any detectable *all-trans*, ß bands in the near UV range [42], points out comparable *1*3-*cis*/*all*-*trans* molar ratios in both, DA and LA samples (Fig 3A and 3B). In the presence of high concentration of KCl, however, LA-DA difference spectra display a negative band at about 513 nm, a positive one at 589 nm, and low intensity ß bands at about 390 nm and 434 nm. Similar spectral features of the two mutants with the difference spectrum of WT (Fig 3C), suggest that the retinal isomers and the conformation of the RBP, engaged in the position of the absorption maximum, are analogous.

#### Isomeric Composition of the Retinal Chromophore in Dark- and Light-Adapted Samples of Multiple EC Glu Mutants

Further, we analyzed the isomer composition of the mutants, by extracting the retinal from DA and LA samples with low (150 mM) and high (1.5 M) concentration of KCl. The analysis of HPLC data reveals that while the *all-trans* isomer constitutes at about 43% of the total retinal pool of the WT bR_DA_ samples, it is the dominant isomer in 2Glu_DA_ (67%), 3Glu_DA_ (63%) and 4Glu_DA_ (62%) samples with low KCl concentration. These findings are rational, considering that E194Q_DA_ sample exhibits about 86% of the *all-trans isomer* [29]. At low KCl concentration, however light-adapted mutant samples show a reduced content of the *all-trans*, decreasing in the following order: bR_LA_ (99%) > E194Q_LA_ (94%) > 2Glu_LA_ (86%) >3Glu_LA_ (78%) > 4Glu_LA_ (67%). Furthermore, we noted that in dark-, but not in light-adapted samples, the amount of the *all-trans*, extracted from the retinal pool is salt-dependent. For example, in 1.5 M KCl the *all-trans* extracted from 3Glu_DA_ sample is at about 47%, that is comparable with bR_DA_ (43%) sample. In light-adapted 3Glu_LA_ and high (1.5 M) KCl however, the *all-trans* retinal constitutes at about 71%. These last values are close to those obtained for 3Glu_LA_ (78%) in low (150 mM) KCl concentration. Most likely, the lower amount of the *all-trans* in 3Glu_LA_ sample, compared with that of bR_LA_ (99%) is due to the formation of *9 cis* and *11 cis* isomers found in the retinal pool of the mutant.

### Chloride-Specific Conformations and Thermal Stability of Multiple EC Glu Mutants

#### Visible—Circular dichroism Spectra

**Fig 4:**
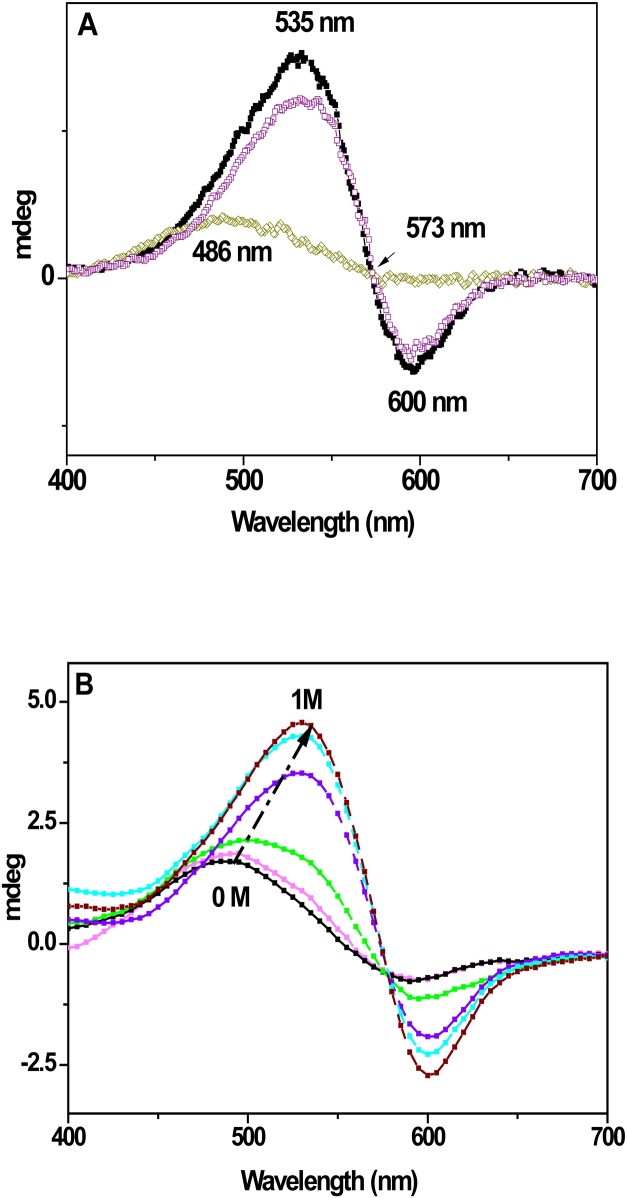
Circular dichroism (CD) experiments in the visible spectral region. (A) Vis-CD spectra of WT (■), E194Q (□) and 2Glu (□) in water, pH 7.0. The spectra of E9Q, E74Q and E204Q single mutants (not shown) are identical with those of E194Q. The CD spectra of WT and of all single mutants reveal the well-known biphasic bands (535/600 nm) of the retinal chromophore, while the multiple mutants show only one band (at about 468 nm). (B) Representative Vis–CD spectra of 3Glu in the presence of increasing (by 0.2 M) concentrations of KCl (pH 6.0). Arrows indicate the direction of spectral changes with KCl increases. Upon addition of KCl, the monophasic spectrum is converted to a biphasic, indicative for conformational changes in the retinal binding pocket.

To track down eventual alteration of the 2D array of the mutated bR, we employed Visible Circular Dichroism (Vis-CD), known as a sensitive spectroscopic tool for analyzing the structural integrity of bR molecules in PM [43,44]. Vis-CD spectrum of WT consists of two asymmetric biphasic lobes at 535 (+) and 600 (-) nm, generated by exciton coupling among the retinal chromophores within bR trimer [44]. Similarly, all single EC mutants exhibit biphasic CD spectra with a crossover at about 573 nm. In contrast, the CD spectra of the multiple mutants exhibit a single positive band at ~486 nm (Fig 4A). Furthermore, similarly to the chloride dependence of the absorption spectra (Fig 1), CD spectra of the mutants undergo mono to bi-phase transformation upon titration with KCl. Moreover, the rise of the intensities of the positive and negative lobes with KCl, accompanying these spectral transformations suggests strengthening of exciton coupling between the retinal chromophores (Fig 4B). Curve fitting of the ellipticity changes at 530 nm vs KCl concentration give apparent dissociation constants (K_d_) of 0.22 ± 0.04 M, 0.47±0.03M, 0.31 ±0.13 M for 2Glu, 3 Glu and 4Glu mutants, respectively. These CD affinity constants closely correlates with K_d_ deduced from the absorption maxima.

#### Thermal stability of inter-trimer chromophore interactions

**Fig 5:**
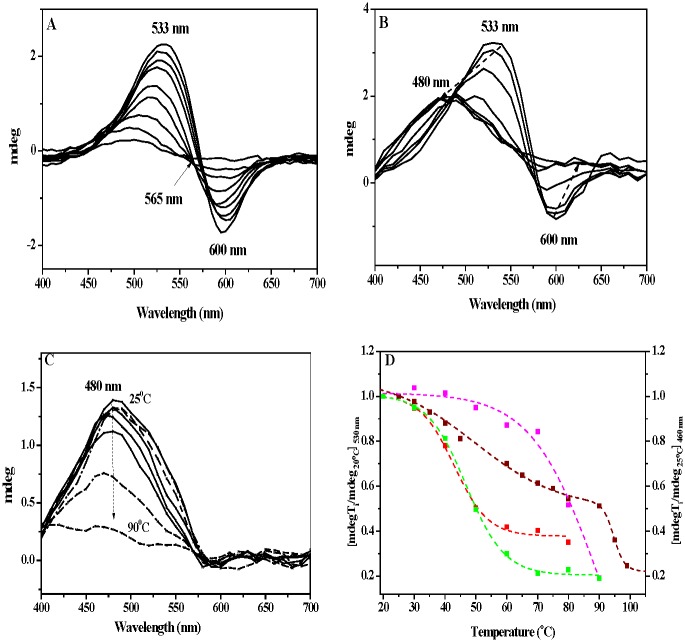
Thermal stability of inter-trimer chromophore interactions CD data. (A) Temperature-dependent Visible–CD spectra of WT in water, pH 6.5. (B) Representative spectra of 3Glu in 1 M KCl, pH 6.5. (C) Representative spectra of 3Glu in water, pH 6.5. (D) Plots of the ratio of the ellipticity at any temperature to that at 20°C, measured at 530 nm for WT(■), 3Glu (■) and 2Glu (■) in 1 M KCl and at 460 nm for 3Glu (■) in water, pH 6.5. The dotted lines represent the best fit of the experimental data, applying non-linear fit with single sigmoid Boltzmann function for 2Glu and 3Glu. The curve for WT shows bi-phase temperature dependence and was fitted to a sum of two sigmoid functions, applying the Boltzmann equation.

Next, we studied the thermal stability of protein arrays by monitoring inter-trimeric exciton chromophore coupling over the temperature. In WT, on an increase in the temperature, magnitudes of the positive (at 533 nm) and negative (at 600 nm) lobes decrease gradually, accompanied with blue shift of the lobes (Fig 5A). Consequently, above 50°C the bands shift to 521 (+) and 591 (-) nm, with an isosbestic point at 565 nm and above 80°C, WT CD spectra display a single band at 478 nm. In 1 M KCl, the CD spectra of the mutants (Fig 5B) are similar to those of WT, suggesting that mutated proteins go through similar conformational changes upon heating. However, analysis of spectral data shows that two-state thermal transitions from exciton coupling of the chromophores (biphasic CD) to loss of the exciton coupling (monophasic CD) occur at different temperatures in the mutants (Fig 5B). Upon an increase in temperature the ellipticity of the bands at 533(+) and 600(-) nm drop exponentially, and above 50°C the spectra consist of a single band at about 480 nm (Fig 5B). At room temperature, unlike the CD spectra of WT (Fig 4A) and mutants in 1 M KCl (Fig 4B), in water or at low salt concentration the CD spectra of the mutants consist of a single band at about 480 nm, suggesting a lack of exciton coupling among the retinal chromophores within the bR trimer [44]. Furthermore, the intensity of the 480 nm band diminishes with temperature increase (Fig 4B) and above 65°C the CD signal exponentially declines, due to denaturation of the protein and sample aggregation. Plots of intensities changes of exciton CD bands at 530 nm versus temperature for WT, 2Glu and 3Glu mutant are shown on Fig 5D. The curve fittings of the data in 1 M KCl give mid-transition temperatures (T_m_) for intermolecular exciton coupling between the chromophores at about 46.9°C and 48.3°C for 2Glu and 3Glu mutants, respectively. Furthermore, the sigmoid curve shapes with a single inflection point, corresponding to a melting temperature (T_m_) of protein-protein contacts, suggests a cooperative process, in which association/dissociation between bR monomers occurs in the mutants. From the plot of ellipticity changes at 530 nm for the WT, two transitions can be deduced, one for coupling-decoupling with a T_m_ at about 50°C, and another for protein unfolding with a T_m_ at about 95°C. The thermal stability of 3Glu in conditions of weak exciton chromophore interactions was studied following the ellipticity changes at 460 nm (Fig 5D, right y axis). The curve fittings of the data for 3Glu mutant in H_2_O gave a T_m_ around 80°C. It is worth to mention that the values for melting transition temperatures for WT and 3Glu in water are in close agreement with the corresponding T_m_ values obtained from the DSC thermograms (see for details the text below). The reversibility of inter-trimer chromophore interactions was tested by cooling the samples back to the starting temperature. We found that CD spectral features were partially reversible for WT samples and for the mutants in 1 M KCl, but not in water.

#### Thermal reversible transitions of multiple EC Glu mutants. Differential scanning calorimetry

**Fig 6:**
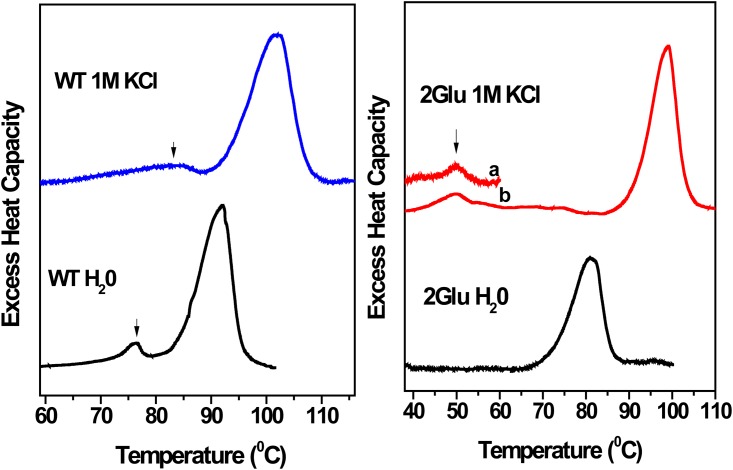
Thermal unfolding experiments. DSC data.Differential scanning calorimetric traces for WT (left panel) and for 2Glu (right panel) recorded in water (black) and in 1 M KCl (colored line) at pH 7.0. a) First scan up to 65°C; b) Second scan of the same sample after cooling down the sample. Scans were taken at scanning rate of 1 K·min^-1^. The DSC data were corrected for the instrumental and chemical base lines and analyzed as explained in Materials and Methods.

As we reported earlier, DSC thermograms of all multiple Glu mutants lack the reversible transition peak, assigned to disordering of WT paracrystalline array in purple membranes [26,35]. Considering the recovery of the bilobe pattern of CD spectra of the mutants with KCl, we further examined whether the reversible DSC transition is also chloride-dependend. Fig 6 shows DSC scans of WT and 2Glu mutant in water and 1 M KCl solutions. DSC thermogram of 2Glu in water displays only one transition peak at about 85°C, in agreement with our previously reported data [26]. However, the thermogram of the mutant in 1 M KCl clearly resolves two transition peaks, one occurring at about 52°C and a second one at 98°C. The first small peak corresponds to the WT reversible transition, as revealed by rescanning of the sample, while the second one at higher temperature depicts the irreversible thermal unfolding of the mutant. Table 1 summarizes the calorimetric parameters for WT and multiple EC Glu mutants. In agreement with the data previously reported [26], in water the mutants exhibit lack of the reversible transition and lower T_m_ of the irreversible transition, compared to WT. In 1 M KCl, a most intriguing feature of the thermograms of the mutants is the recovery of the WT-like reversible transition, though at lower T_m_ compared to WT. Furthermore, with regard to water, T_m_'s of the main irreversible transition shift to higher temperatures, close to the WT T_m_ and the associated enthalpies increase by ~20%, comparable with the unfolding enthalpy of WT. On the other hand, despite having higher T_m_ of protein denaturation, the unfolding enthalpies have lower values in comparison to WT unfolding in 1 M KCl.

**Table 1:** Calorimetric parameters from thermal unfolding of WT, 2Glu and 3Glu, measured in water and 1 M KCl, at pH 7.0 by DSC.

Sample	WTH_2_O	WTKCl, 1 M	2GluH_2_O	2GluKCl, 1 M	3GluH_2_O	3GluKCl, 1 M
^a^Main transition*(T_m_, °C)	92.1	102.0	84.9	98.9	84.6	93.0
Pre-transition*(T_m_, °C)	76.2	85.0	N.D.	49.9	N.D.	54.6
^b^ΔH (KJ/mol)	364.36	473.7	176.0	207.0	163.0	220.4

^a^Temperature of the maximum of the heat capacity trace

^b^Calorimetric enthalpies were calculated by using the NanoAnalyse software.

*T_m_ values for WT and the mutants in water are in close agreement with our previously reported data. [24,26,27,36]. Small differences for T_m_ can be accounted for different scanning rates used, knowing that transitions are kinetically controlled.

N.D., not determined.

### Molecular Dynamics of Chloride Binding in Multiple EC Glu Mutants

**Fig 7:**
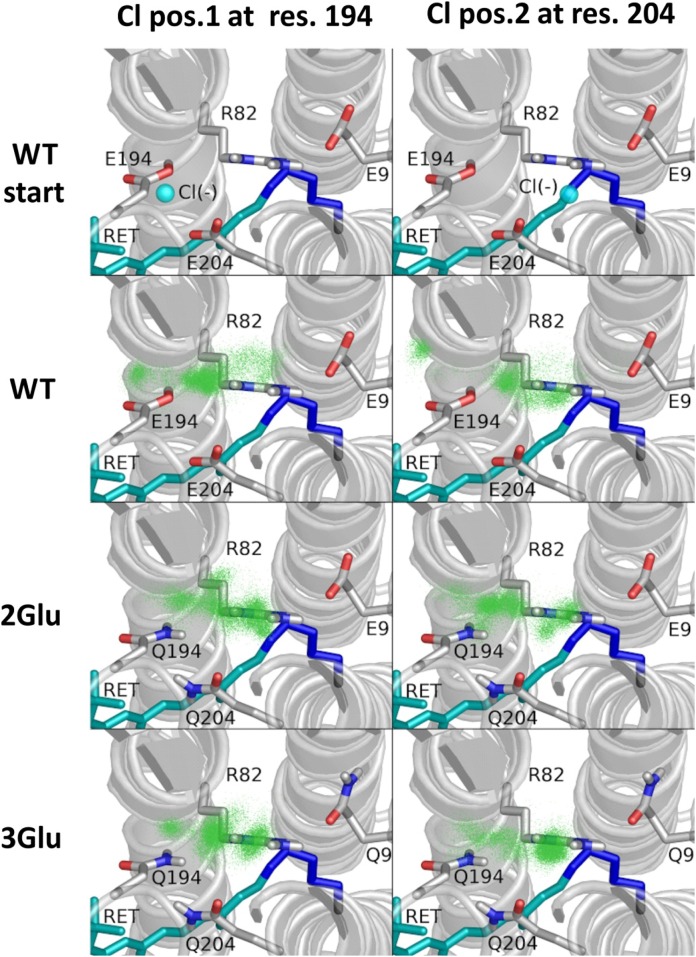
Positions of chloride ion close to the proton release group in MD simulations. Starting points and summary positions for two different locations are shown in columns. Starting positions are exemplified in WT structure (top row). For subsequent rows: in green summary locations of Cl^-^ ion (from 9 monomers of bR) in WT, 2Glu and 3Glu during 100 ns MD simulation.

**Fig 8:**
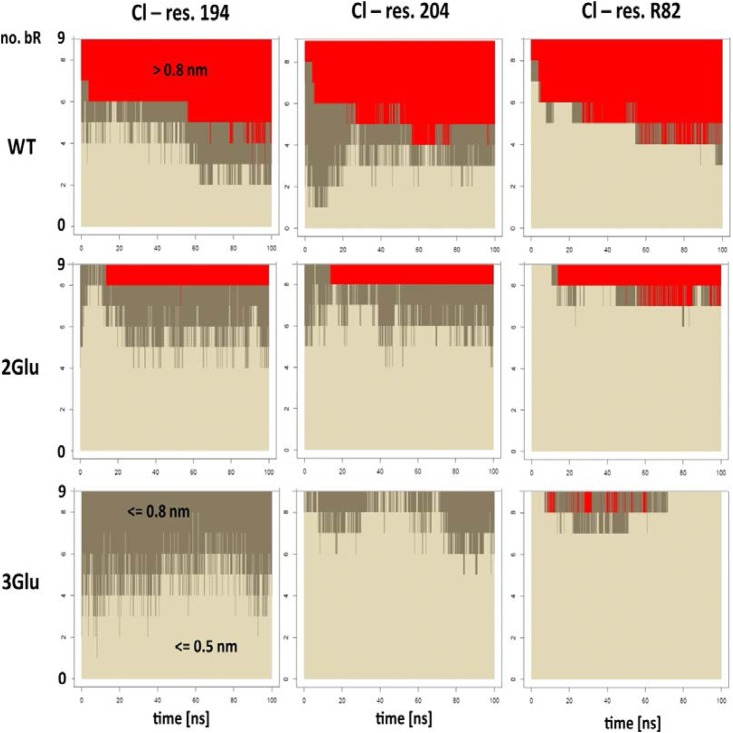
Distances of Cl ion from particular residues, Glu/Gln194, Glu/Gln204 and Arg82, during 100 ns MD simulations for each of nine bR molecules. Colors indicate distance ranges: light gray < = 0.5 nm (binding area), dark gray < = 0.8 nm (proximity area), and red > 0.8 nm (escape).

Further evidence for anion-specific binding of the Cl ion within mutated bR comes from MD simulations data. To examine an eventual interaction between Cl^-^ and bR monomer and to evaluate whether it enters the interior of bR, we performed two sets of simulations. In the first set, in 2M NaCl, the anion coming from the bulk was expected to occupy a space in the proximity of retinal. However, throughout the 150 ns of MD simulation was not observed any apparent entering of Cl^-^ into the inner bR space, neither for WT nor for 3Glu mutant. A possible reason could be that the time span was not long enough or alternatively that the manner by which Cl^-^ restores the WT behavior is different from that expected. In the second set of simulations, the Cl^-^ anion was placed in two alternative positions: a first position, near Glu/Gln194 side chain and in a second position, next to Glu/Gln204 side chain (Fig 7 top row). During the course of 100 ns MD simulations, we observed that the Cl^-^ escapes from half of WT monomers. It is worth noting that usually the escape event occurred during the equilibration phase, which lasted 2.5 ns. In contrast to that, in both 2Glu and 3Glu mutants the Cl^-^ appears to hold within the inner hydrogen bonds network, consisting of water, aspartate and glutamate residues (Fig 7). In most of the cases, the anion relocates from its initial position and form an electrostatic interaction with R82 residue (S1–S6 Figs). We found a very clear trend in our 100 ns simulations for a weak Cl^-^ binding to WT (presence in binding area and partly in proximity area in about 40% cases) and high binding to 2Glu (75%) and 3Glu (90%) mutants (Fig 8).

## Discussion

Our previous studies have shown that 2Glu, 3Glu and 4Glu mutants of bR exhibit remarkably conformational and stability alterations compared to WT. Mainly, in water and in low ionic strength media, these multiple Glu mutants comprise blue—shifted visible absorption maxima, abnormal dark to light adaptation and disordering of the paracrystalline arrays of mutated bR molecules [24,26,36].

In this study, to address the molecular nature of the conformational and stability alterations, generated upon multiple replacement of the negatively charged EC Glu with Gln, we performed experiments varying solvent ionic strength and composition. Absorption spectroscopy experiments reveal that blue-shifted λ _max_ of multiple Glu mutants (up to 60 nm in comparison with WT) can be fine-tuned by varying medium´s ionic strength (Fig 1). Apparently, as shown by the experiments carried out with salts composed of ions of different sizes and charges, this spectral phenomenon is due to halide (Cl^-^, I^-^) anion presence in the media, which most likely binds to specific sites in the mutated bRs (Fig 1C). Spectral titration data show that a half-maximum shift of λ_max_ is reached at millimolar concentrations of KCl in the solution with apparent dissociation constants (K_d_) at 0.16 M, 0.38 M and 0.22 M KCl for 2Glu, 3Glu and 4Glu mutants, respectively. These values imply for specific binding of the Cl^-^ in close proximity to the retinal binding site. This assumption is consistent with the demonstrated dependency of apparent SB pKa on KCl concentration, which at low salt concentration are higher by 0.5 pH units than the WT pKa, implying a more stable chromophore-binding site, and at high concentration are similar to the WT pKa. Moreover, the analogous curves obtained for all three mutants point to similar conformations of the SB environment (Fig 2C). The manifested couplings between the apparent SB pKa, the λ_max_ and the chloride concentration in the solution suggest that Cl^-^ contributes to a Schiff base counter-ion charge and consequently modulates the conformation of RBP and the spectral properties of the mutants.

Next, we identified the cause for the previously reported abnormal dark to light adaptation in these mutants [24,45]. Analysis of UV-Vis difference spectra revealed that *13-cis* to *all–trans* isomerization of unphotolysed bR, triggered by light in WT is disabled in the multiple EC Glu mutants at low (0.15 mM—0.4 M) KCl concentration, but it occurs when the solvent ionic strength exceeds some threshold value (above 1 M). Furthermore, we found that the concentration of KCl needed for the recovery of the intrinsic WT dark to light adaptation of the mutants differs from that needed for the restoration of WT- like λ _max_ [35,36]. These observations raised the possibility that the anion occupies more than one binding site within multiple Glu mutants. Apparently, binding of one Cl^-^ near RBP changes the electrostatic balance of the SB counter ion, and thus it becomes involved in the color control and alkaline hydrolysis of the SB, while binding of different Cl^-^ restores *13-cis* to *all-trans* isomerization of mutated bRs in wild- type fashion.

The CD experiments indicate that the conformational alterations, produced by multiple substitutions of the negatively charged EC Glu residues for Gln, are not restricted only to the retinal vicinity. In water, a single positive peak at 500 nm, characterizing the CD spectra of the multiple mutants indicates weakened exciton coupling between the retinal chromophores. The addition of KCl reestablishes WT-like bilobe features of CD spectra (Fig 4B), with binding affinity close to that calculated for the restoration of WT-like absorption maximum. These findings imply that the specific binding of Cl^-^ can modulate loosened protein-protein interactions in the mutants and reestablish the proper WT-like packing of mutated bR monomers in the membrane.

Thermal CD and DSC data served as diagnostic tools for evaluation of the stability of inter-trimer chromophore interactions and of the tertiary structure to temperature stress. The thermodynamic parameters provide a direct energetic picture of protein unfolding, known to occur through exposure of non-polar and polar groups to water, breaking of a number of hydrogen bonds and salt bridges with temperature increase [46]. In addition, the DSC data for WT reports on the strength of the interactions, maintaining the paracrystalline array and tertiary structure [14]. In water, all multiple Glu mutants show greatly affected thermodynamic parameters including lost of the reversible transitions, lower melting temperatures and unfolding enthalpies, with a destabilization effect up to 200 KJ/mol, as compared to WT (Table 1). Actually, as we reported previously, lower cooperativity unfolding and lower T_m_ of reversible and main transitions were already observed for the single mutants, E194Q, E204Q, and especially E9Q (with lower T_m_ pre-transition by 20°C), compared to WT [26]. Altogether, these findings imply that the side chains of these Glu residues, located close to the EC surface participate in interactions that maintain 2D paracrystalline array and tertiary stability of the WT bR. The DSC data presented here clearly show that the specific binding of Cl^-^ reestablishes these interactions and the protein integrity of mutated bRs. In 1 M KCl, DSC data display reappearance of the reversible thermal transition, higher T_m_ of denaturation and an increase in the enthalpy of unfolding of the mutants. Though the melting temperatures, T_m_'s, of mutants get closer to the WT T_m_, the enthalpies keep lower values, compared with WT. These data indicate that the mutated bR need less energy for their unfolding, most likely due to loss of some hydrogen bond and salt bridge interactions upon mutagenesis. It is worth to mention a close proximity between T_m_ of calorimetric reversible transitions, reporting disordering of the 2D-array of 2Glu and 3Glu in 1 M KCl (Table 1) with T_m_ of thermal dissociation of inter-trimer exciton chromophore coupling, deduced from CD data (Fig 5D).

The analysis of the experimental data, directs us to the question of how each of the four glutamates contributes to the maintenance of the ground state bR structure. Our findings essentially point out that the multiple mutants, containing both E194Q and E204Q mutations experience blue-shifted maxima, altered protein-protein contacts and loss of paracrystalline array, alterations not found in any of the single EC Glu mutants [47–49]. Apparently, the simultaneous loss of the ionizable side chains of the two glutamates is the major cause for the observed conformational and stability perturbations, described for 2Glu, 3Glu and 4Glu mutants. The only exception corresponds to the abnormal thermal isomerization of the mutants, which is actually due to the substitution of Glu194 for Gln. As we reported recently, Glu194 is involved into *all-trans* to *13-cis* thermal isomerization of unphotolysed bR [29].

**Fig 9:**
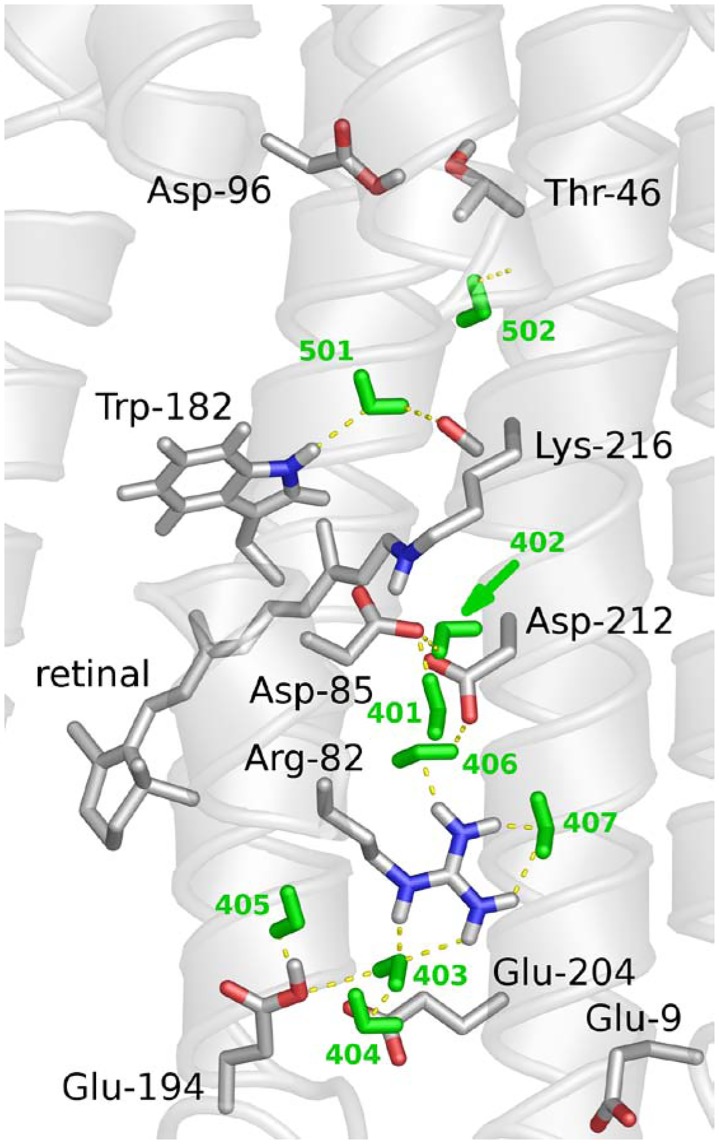
X ray structure of extracellular side of ground state bacteriorhodopsin, Protein Data Bank entry 1C3W [12,50]. Added and optimized are hydrogen atoms, presenting the continuous hydrogen bonding network, connecting proton release group with protein active site. Arg82 connects with Glu194 through water molecules W403 and W404 (green), while the side chains of Glu194 and Glu204 form a direct hydrogen bond. In addition, Arg82 connects to Asp85 through Trp 402, Asp212, Tyr57 and Trp407.

Though all three multiple mutants experience similar conformational and structural stability alterations, we noted an apparent difference of affinity constants for color regulation and retinal exciton interactions between 2Glu and 3Glu mutants. These data indicate that, besides Glu194 and Glu204, also Glu9 may participate in interactions defining the color and retinal isomerization, as well as the tertiary protein stabilization. The last assumption is in accordance with the lower melting temperature and less cooperative unfolding of the single E9Q mutant [26]. In fact, the X-ray structure of the ground state bR discloses inter-helical hydrogen bonds between Glu9 and Tyr79 [12]. Furthermore, EC water molecules, W404 and W405, are stabilized by hydrogen bonding with Glu194–OE2 and Glu204–OE2, and by another hydrogen bond to two peptide groups of Tyr79 [50] (Fig 9). X-ray mapped hydrogen bond interactions of residue 9 further reinforces likely contribution of Gln9 to the spectral phenomenon and conformational alterations, generated by the simultaneous mutation of both residues, Glu204 and Glu194 for Gln. In contrast, a comparison of the UV-Vis absorption and CD spectral data for 3Glu and 4Glu mutants suggests a modest influence of Glu74 on the spectral properties and intermolecular contacts, in accord with previously reported data for E74Q, claiming WT-like behavior of this mutant [26, 48].

In the ground state of bR the cluster, formed by Glu194, Glu204 and a water molecule associated with the latter two residues, has a net charge of −1 [51]. The crucial role of this cluster, serving as proton release group (PRG) for bacteriorhodopsin functioning is well established [52]. A single replacement of Glu194 or Glu204 with a neutral Gln affects protein functionality, resulting in altered photocycle and late proton release. Intriguingly, however neither E194Q nor E204Q mutants exhibit conformational and structural alterations, as those observed upon the simultaneous E194Q/E204Q mutation [48,49]. Thus, a question arises about molecular mechanisms responsible for these alterations, requiring a simultaneous elimination of the negatively charged side chain of these Glu residues.

High-resolution map discloses seven internal water molecules and interacting residues in the EC site of bR [12,50]. Furthermore, an extensive H-bonded network links the terminal PRG, near the extracellular surface to the Schiff base via Arg82, W406, Asp85, W402, and additional waters (Fig 9). From a structural perspective, it is reasonable to expect that the loss of the negatively charged cluster, formed by the two glutamates, Glu194 and Glu204, will disrupt some of the specific interactions, primarily the hydrogen bonds, connecting the latter two residues and associated water. Certainly, this loss may cause further disordering of the continuous H-bond network and dislocation of certain key residues, interconnected through this network. Consequently, the electrostatic balance of the SB counter ion will change and affect the spectral properties of mutated bR. Recent MD calculations support this view, demonstrating that simultaneous mutations of Glu204 and Glu194 result in a loss of half of the hydrogen bonds with water molecules [24].

The second effect of simultaneous mutation of EC Glu residues relates to distortion of the intermolecular protein-protein and lipid-protein interactions, ensuring the thermal stability of 2D array of bR. High-resolution structures of WT have revealed that bR trimers are stabilized by interactions between B and D helices and, to a minor degree, between helices A and F of the adjacent bR molecules forming the trimer [11]. Furthermore, due to the continuous H-bond network, the EC side of WT bR exhibits more rigid dynamics than the CP site [53]. Recent MD simulations performed on the 3Glu reported overall destabilization of the trimer arrays of the mutant, consisting of more flexible and less stable EC area, spanning from helix B to the center of helix E, including the whole β-sheet loop in comparison to the WT structure [24].

Here we report solid experimental evidence that the conformational and stability perturbations, imposed with the loss of the negatively charged cluster can be successfully compensated with the specific Cl^-^ binding. The small chloride anion, known as a good hydrogen-bond acceptor and ion-pair partner is generally recognized as counter ion directly interacting with proteins [54]. MD simulations of 2Glu and 3Glu mutants (Fig 7 and S1–S6 Figs) validate our experimental findings, indicating high affinity binding of Cl^-^ to Arg82 and the two Gln204 and Gln194 residues, thus turning these mutants into anion-sensitive pigments.

The specific binding of Cl^-^ near to the three key residues of the EC region reconstitute the conformation and tertiary structure stability of mutated bR in WT-like fashion. The wild type like properties of these mutants indicates that Cl^-^ binding efficiently coordinates distorted hydrogen bonding network into EC region. The binding of Cl^-^ affects not only the ground state of mutated proteins, but also the functional intermediates, responsible for conformational switching under illumination. As previously reported, the accumulation of cytoplasmic N and O photo intermediates is Cl^-^ dependent for E194Q and multiple EC Glu mutants [49].

In conclusion, our results unveil that distortion of H-bond interactions between key functional residues and EC water molecules can severely affect the conformation stability and 2D array of bR. These findings challenge the view that hydrogen bonds play only small and localized role in membrane protein stability [55]. Molecular forces that contribute to protein structure and stability are important for understanding the function of retinal photoreceptors, as it was recently reported H-bond network regulates the optical properties of short-wavelength-sensitive visual Rhodopsin [56].

## Supporting Information

S1 Appendix provides Molecular Dynamics Methodology with References. (PDF)

S1 Fig Locations of Cl^-^ ion in each of nine WT monomers of bR during 100 ns MD simulation. The initial position of Cl^-^ was close to residue Glu194. Color range blue-white-red indicates temporal position of ion during simulation (blue = initial position). Lack of red dots indicates escape of the ion.
(PDF)

S2 Fig Locations of Cl^-^ ion in each of nine WT monomers of bR during 100 ns MD simulation. The initial position of Cl^-^ was close to residue Glu204. Color range blue-white-red indicates temporal position of ion during simulation (blue = initial position). Lack of red dots indicates escape of the ion.
(PDF)

S3 Fig Locations of Cl^-^ ion in each of nine 2Glu monomers of bR during 100 ns MD simulation. Color range blue-white-red indicates temporal position of ion during simulation (blue = initial position). The initial position of Cl^-^ was close to residue Gln194.
(PDF)

S4 Fig Locations of Cl^-^ ion in each of nine 2Glu monomers of bR during 100 ns MD simulation. Color range blue-white-red indicates temporal position of ion during simulation (blue = initial position). The initial position of Cl^-^ was close to residue Gln204.
(PDF)

S5 Fig Locations of Cl^-^ ion in each of nine 3Glu monomers of bR during 100 ns MD simulation. Color range blue-white-red indicates temporal position of ion during simulation (blue = initial position). The initial position of Cl^-^ was close to residue Gln194.
(PDF)

S6 Fig Locations of Cl^-^ ion in each of nine 2Glu monomers of bR during 100 ns MD simulation. Color range blue-white-red indicates temporal position of ion during simulation (blue = initial position). The initial position of Cl^-^ was close to residue Gln204.
(PDF)

S1 Table Description of MD Simulation Sets. (PDF)
