# Supplementary material for: Identification of Specific Effect of Chloride on the Spectral Properties and Structural Stability of Multiple Extracellular Glutamic Acid Mutants of Bacteriorhodopsin
Source: PLoS One. 2016 Sep 22;11(9):e0162952. doi: 10.1371/journal.pone.0162952 (PMC5033488; doi:10.1371/journal.pone.0162952)
Supplement: S1 Appendix — (PDF) [file pone.0162952.s001.pdf]

S1 Appendix

Identification of Specific Effect of Chloride on the Spectral Properties and Structural Stability of Multiple Extracellular Glutamic Acid Mutants of Bacteriorhodopsin

Tzvetana Lazarova[1], Krzysztof Mlynarczyk[2], Enric Querol[3], Boris Tenchov[4], Slawomir Filipek[2], Esteve Padrós[1]

[1]Unitat de Biofísica, Departament de Bioquímica i de Biologia Molecular, Facultat de Medicina, and Centre d'Estudis en Biofísica, Universitat Autònoma de Barcelona, Barcelona, Spain

[2]Faculty of Chemistry, Biological and Chemical Research Centre, University of Warsaw, Warsaw, Poland

[3]Institut de Biomedicina i Biotecnologia, Universitat Autònoma de Barcelona, Barcelona, Spain

[4]Department of Medical Physics and Biophysics, Faculty of Medicine, Medical University – Sofia, Sofia, Bulgaria

Supporting Information

Molecular Dynamics Methodology

Composition of lipids for molecular dynamics. The systems investigated in molecular dynamics (MD) simulations included the natural membrane lipids PGP-Me (archaetidylglycerol methylphosphate), which accounted for ~70% of total number of lipids, S-TGA-1 (sulphated triglycosyl diphytanylglycerol), accounting for ~20%, and squalene, ~10%, 63-18-9 of particular lipids, respectively, in a periodic box containing nine bR molecules. The dimensions of the periodic box were: a=10.4 nm, b=10.4 nm, c=16.1 nm, angles: ab=60°, bc=90°, ac=90°. The system was the same as we used for study of structural stability of bR mutants [1].

Molecular dynamics of BR in the purple membrane. The bR trimer, constructed by geometrical operations according to crystallographic data, was subjected to a short energy minimization in the lipid environment and subsequently it was used for the construction of the nonamer. This procedure was followed by the addition of water and ions to a desired concentration. The whole system was further energy minimized

and equilibrated in the NVT ensemble - the equilibration was performed in five stages of different lengths: 10 ps, 5 ps, 5 ps, 5 ps and 10 ps. The first stage involved applying of positional restraints on all heavy atoms, using a force constant ranging from 1000 kJ/mol (in first stage) to 200 kJ/mol (in a last stage) and a temperature of 200 K (first stage) that was raised gradually to the final value of 300 K. NVT equilibration was followed by a sequence of five equilibration runs in the NPT ensemble (10, 5, 5, 5 and 2500 ps, respectively) with restraints limited to protein backbone (200, 150, 100, 50 and 0 kJ/mol), followed by at least 100 ns of production simulation without restraints (details in Table 1). During these simulations the temperature of the membrane, including the protein, and the solvent was coupled independently to a heat bath using the v-rescale thermostat [2] with coupling constant of 0.1 ps at 300 K. The pressure was weakly coupled to a reference pressure of 1 bar using anisotropic Berendsen barostat [3] with a coupling time of 1.0 ps and a compressibility of 4.5×10^{-5} bar^{-1}. MD simulations were performed using an integration time step of 2 fs. The bond lengths and angle of the water molecules were constrained using the SETTLE algorithm [4]. Bond lengths within the protein were constrained using the LINCS algorithm [5, 6]. Water was modeled explicitly using the simple point charge (SPC) model [7]. All simulations were performed employing the united atom GROMOS 43a2 force field with an extension for aliphatic hydrocarbons [8]. Parameters for the head groups of the glycolipids were modeled using the carbohydrate parameters from the GROMOS 45a3 force field [9]. The non-bonded interactions were evaluated using cutoffs of 0.9 nm for short range electrostatic and 1.4 nm for Van der Waals interactions. Long range electrostatic interactions were calculated using Particle-Mesh Ewald summation (PME) [10, 11].

Description of simulation sets. Two sets of simulations were performed. The details are described in S1 Table.

Molecular dynamics of bR in the purple membrane. All simulations of bR nonamer and most of the analyses were performed using the GROMACS v.4 molecular dynamics package [12]. For visualization of trajectories, the VMD program [13] was used. For ion placement determination for set 2, YASARA package was used [14]. The figures were prepared in PyMOL [15]. Data analysis was performed using appropriate tools from Gromacs package, followed by post processing in R package [16].

References

1. Lazarova, T.; Mlynarczyk, K.; Filipek, S.; Kolinski, M.; Wassenaar, T. J.; Querol, E.; Renugopalakrishnan, V.; Viswanathan, S.; Padrós, E. The effect of triple glutamic mutations E9Q/E194Q/E204Q on the structural stability of bacteriorhodopsin. *FEBS Journal* **2014,** 281, 1181-1195.
2. Bussi, G.; Donadio, D.; Parrinello, M. Canonical sampling through velocity rescaling. J. Chem. Phys. 2007, 126, 014101.
3. Berendsen, H. J. C.; Postma, J. P. M.; Vangunsteren, W. F.; Dinola, A.; Haak, J. R. Molecular-Dynamics with Coupling to an External Bath. J. Chem. Phys. 1984, 81, 3684-3690.
4. Miyamoto, S.; Kollman, P. A. Settle - an Analytical Version of the Shake and Rattle Algorithm for Rigid Water Models. J. Comput. Chem. 1992, 13, 952-962.
5. Hess, B.; Bekker, H.; Berendsen, H. J. C.; Fraaije, J. G. E. M. LINCS: A linear constraint solver for molecular simulations. J. Comput. Chem. 1997, 18, 1463-1472.
6. Hess, B. P-LINCS: A parallel linear constraint solver for molecular simulation. J. Chem. Theory Comput. 2008, 4, 116-122.
7. Berendsen, H.; Postma, J.; van Gunsteren, W.; Hermans, J. Interaction Models For Water In Relation To Protein Hydration. In Intermolecular Forces, Pullman, B., Ed. D. Reidel Publishing Company: 1981; pp 331-342.
8. Schuler, L. D.; Daura, X.; Van Gunsteren, W. F. An improved GROMOS96 force field for aliphatic hydrocarbons in the condensed phase. J. Comput. Chem. 2001, 22, 1205-1218.
9. Lins, R. D.; Hunenberger, P. H. A new GROMOS force field for hexopyranose-based carbohydrates. J. Comput. Chem. 2005, 26, 1400-1412.
10. Darden, T.; York, D.; Pedersen, L. Particle Mesh Ewald - an N.Log(N) Method for Ewald Sums in Large Systems. J. Chem. Phys. 1993, 98, 10089-10092.
11. Essmann, U.; Perera, L.; Berkowitz, M. L.; Darden, T.; Lee, H.; Pedersen, L. G. A Smooth Particle Mesh Ewald Method. J. Chem. Phys. 1995, 103, 8577-8593.
12. Pronk, S.; Pall, S.; Schulz, R.; Larsson, P.; Bjelkmar, P.; Apostolov, R.; Shirts, M. R.; Smith, J. C.; Kasson, P. M.; van der Spoel, D.; Hess, B.; Lindahl, E. GROMACS 4.5: a high-throughput and highly parallel open source molecular simulation toolkit. Bioinformatics 2013, 29, 845-854.
13. Humphrey, W.; Dalke, A.; Schulten, K. VMD: Visual molecular dynamics. J. Mol. Graph. 1996, 14, 33-38.
14. Krieger, E.; Koraimann, G.; Vriend, G. Increasing the precision of comparative models with YASARA NOVA--a self-parameterizing force field. Proteins 2002, 47, 393-402.
15. Schrodinger, LLC. The PyMOL Molecular Graphics System, Version 1.3r1. 2010.
16. Team, R. C. R: A Language and Environment for Statistical Computing. Vienna, Austria, 2015.
