# Supplementary figures and images for: Identification of Specific Effect of Chloride on the Spectral Properties and Structural Stability of Multiple Extracellular Glutamic Acid Mutants of Bacteriorhodopsin
Source: PLoS One. 2016 Sep 22;11(9):e0162952. doi: 10.1371/journal.pone.0162952 (PMC5033488; doi:10.1371/journal.pone.0162952)

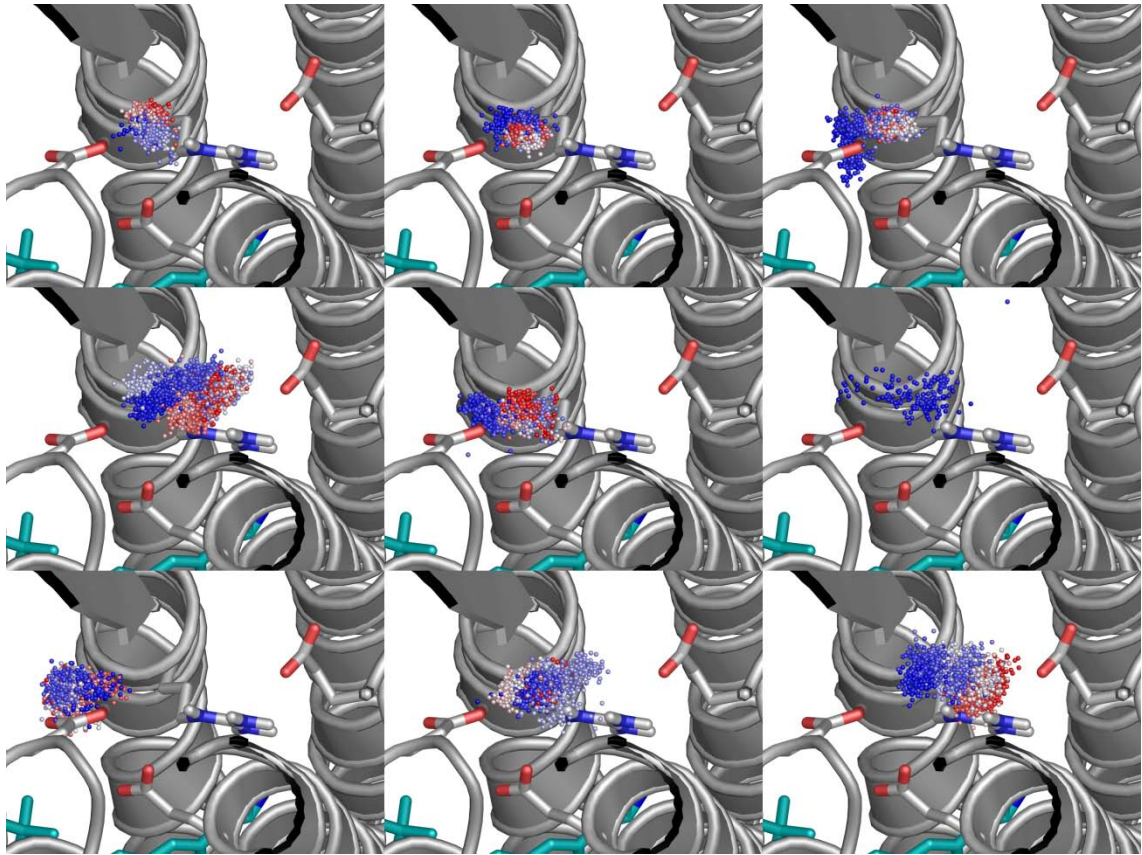

Supplement: S1 Fig — The initial position of Cl- was close to residue Glu194. Color range blue-white-red indicates temporal position of ion during simulation (blue = initial position). Lack of red dots indicates escape of the ion. (PDF) [file pone.0162952.s002.pdf]

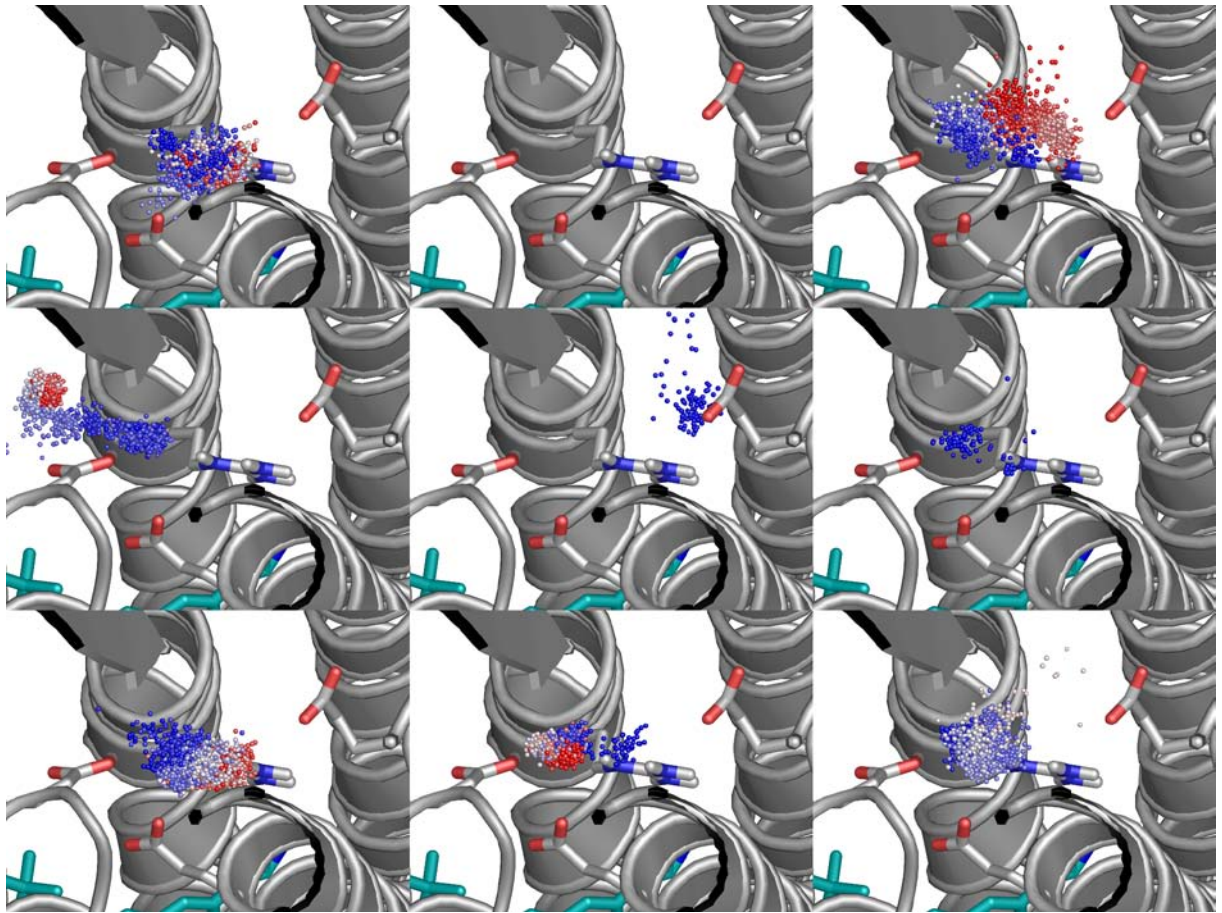

Supplement: S2 Fig — The initial position of Cl- was close to residue Glu204. Color range blue-white-red indicates temporal position of ion during simulation (blue = initial position). Lack of red dots indicates escape of the ion. (PDF) [file pone.0162952.s003.pdf]

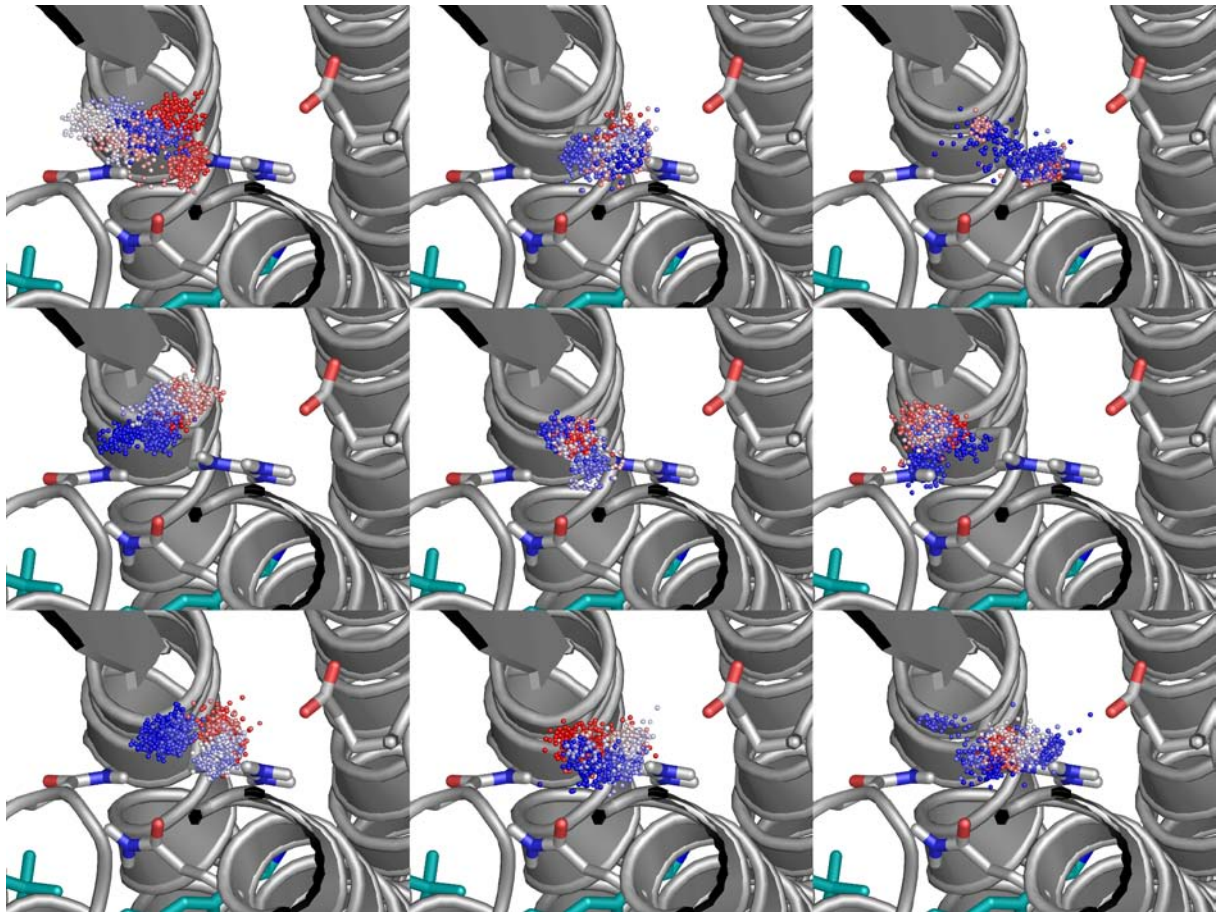

Supplement: S3 Fig — Color range blue-white-red indicates temporal position of ion during simulation (blue = initial position). The initial position of Cl- was close to residue Gln194. (PDF) [file pone.0162952.s004.pdf]

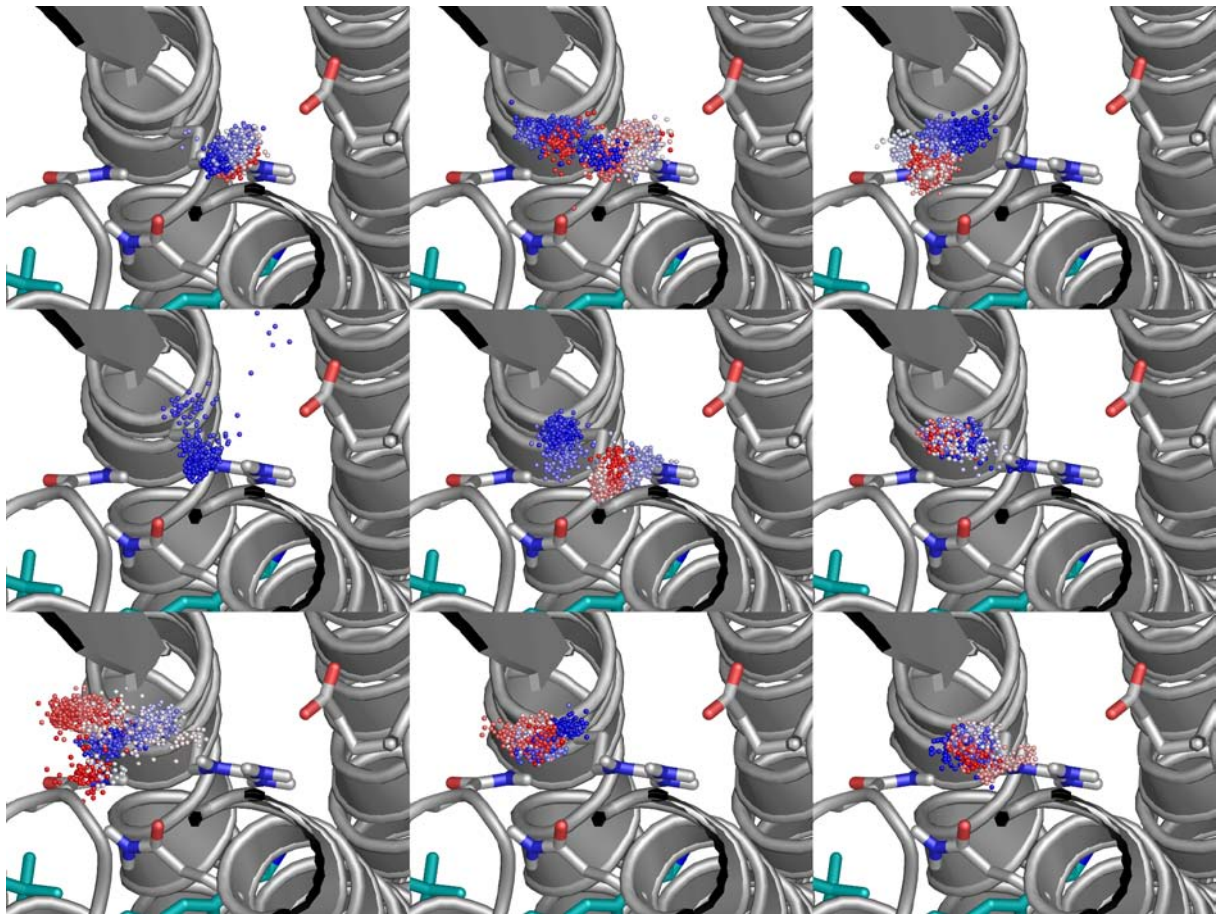

Supplement: S4 Fig — Color range blue-white-red indicates temporal position of ion during simulation (blue = initial position). The initial position of Cl- was close to residue Gln204. (PDF) [file pone.0162952.s005.pdf]

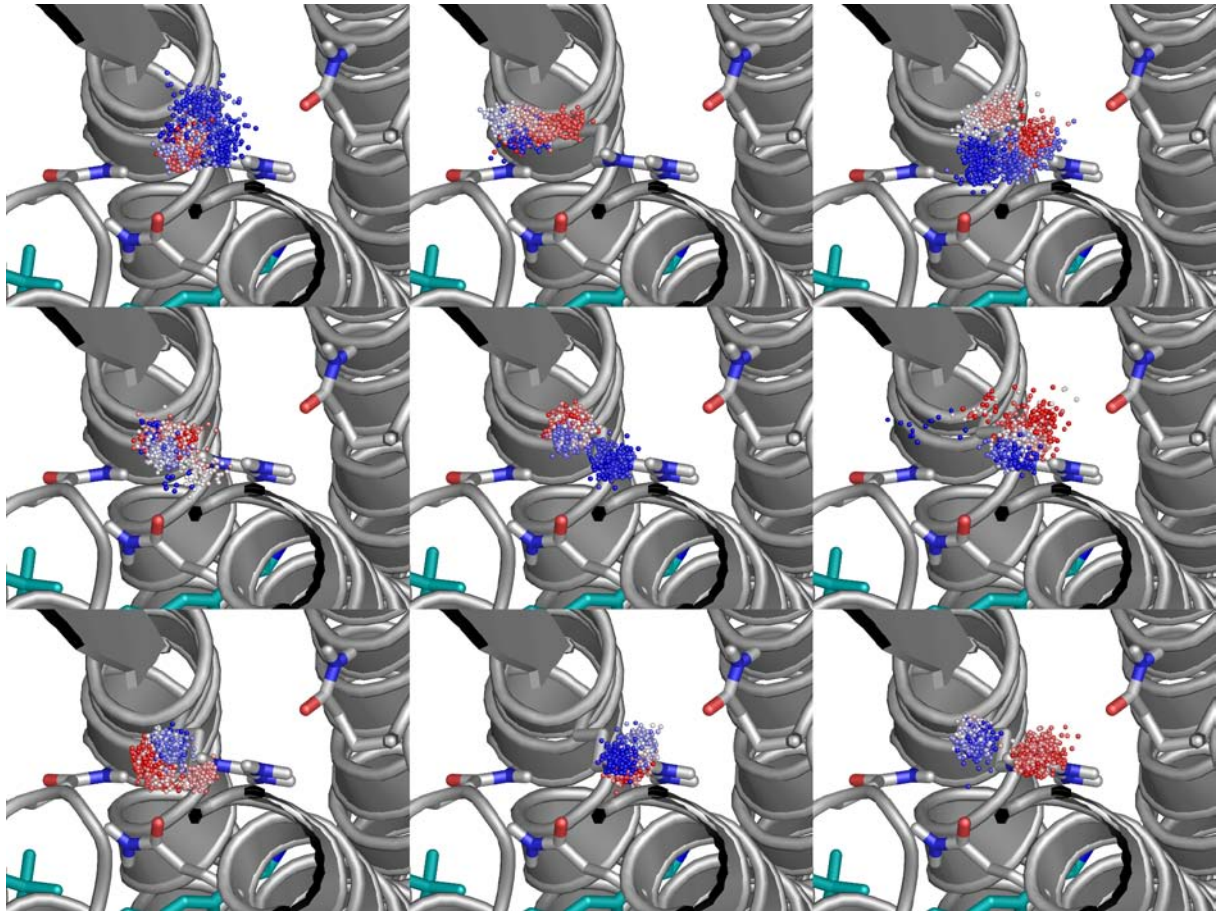

Supplement: S5 Fig — Color range blue-white-red indicates temporal position of ion during simulation (blue = initial position). The initial position of Cl- was close to residue Gln194. (PDF) [file pone.0162952.s006.pdf]

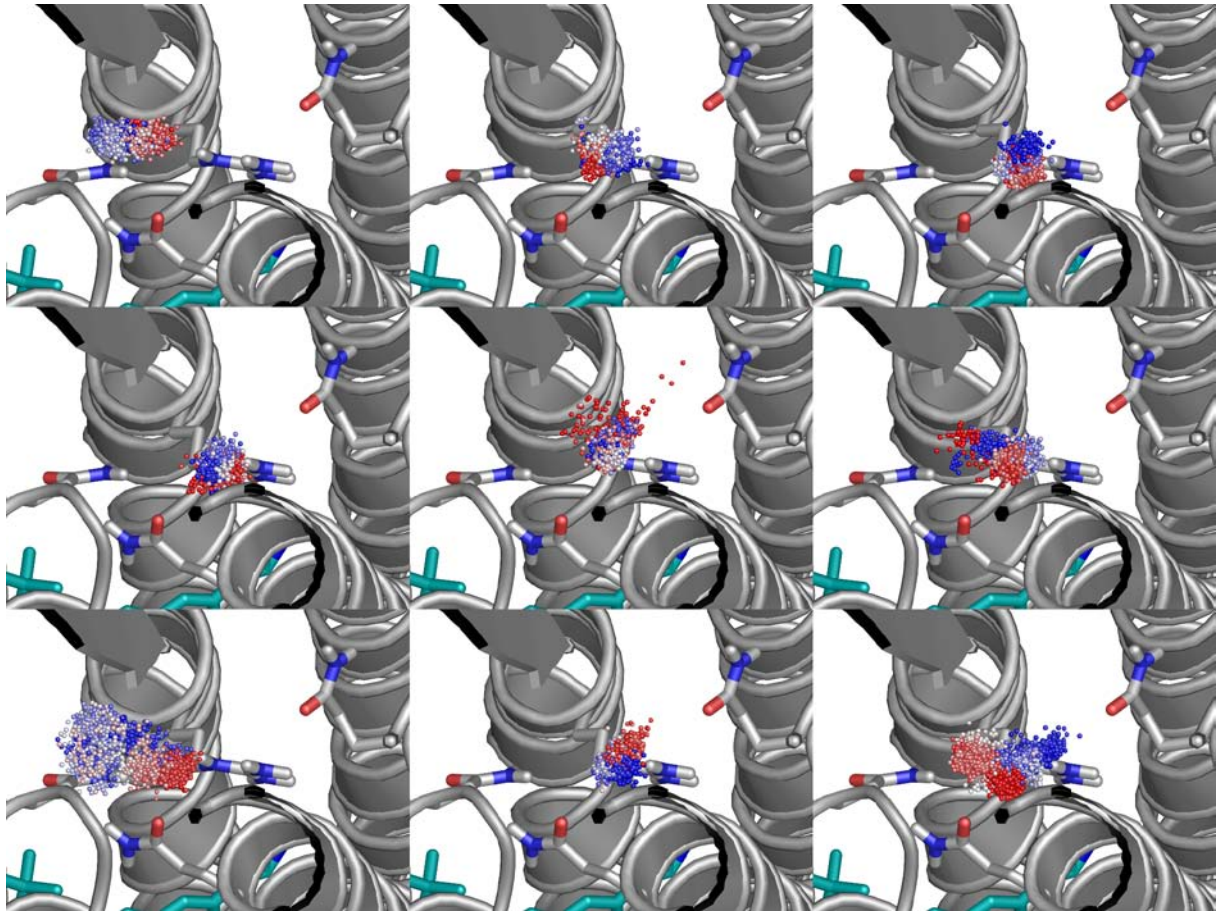

Supplement: S6 Fig — Color range blue-white-red indicates temporal position of ion during simulation (blue = initial position). The initial position of Cl- was close to residue Gln204. (PDF) [file pone.0162952.s007.pdf]
