# Supplementary material for: Identification of Specific Effect of Chloride on the Spectral Properties and Structural Stability of Multiple Extracellular Glutamic Acid Mutants of Bacteriorhodopsin
Source: PLoS One. 2016 Sep 22;11(9):e0162952. doi: 10.1371/journal.pone.0162952 (PMC5033488; doi:10.1371/journal.pone.0162952)
Supplement: S1 Table — (PDF) [file pone.0162952.s008.pdf]

S1Table. The details of performed MD simulations

Set No.	1	2
Protein variants	WT and 3GLU	WT, 2GLU and 3GLU
Goal	Investigation of ion entry path	Determination of the ability of the protein to hold the ion in a predetermined position
Time [ns]	150	100
C_{NaCl} [M]	2	0.15
